# Assessment of Long-Term Degradation of Adsorbents for Direct Air Capture by Ozonolysis

**DOI:** 10.1021/acs.jpcc.4c07054

**Published:** 2024-12-20

**Authors:** Shubham Jamdade, Xuqing Cai, David S. Sholl

**Affiliations:** † School of Chemical & Biomolecular Engineering, Georgia Institute of Technology, Atlanta, Georgia 30332-0100, United States; ‡ Oak Ridge National Laboratory, Oak Ridge, Tennessee 37830, United States

## Abstract

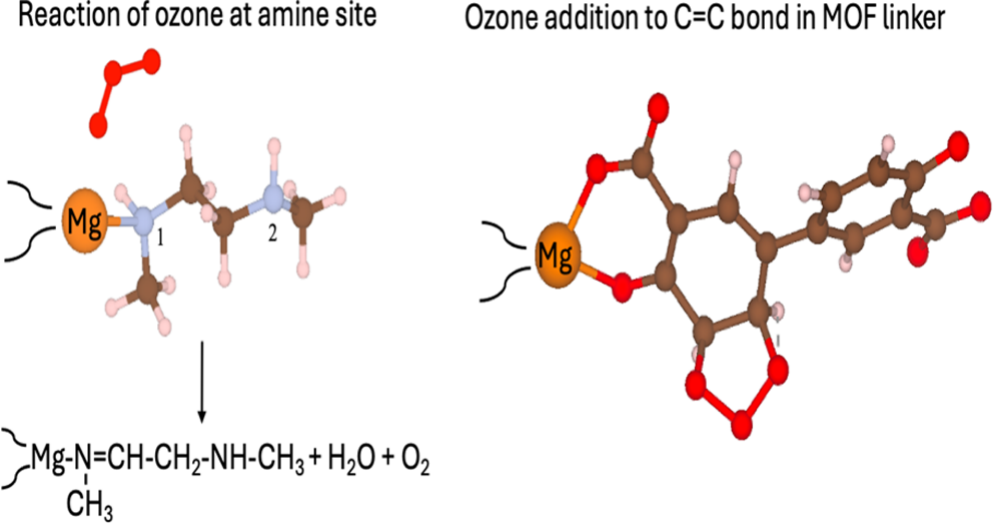

Porous adsorbents are a promising class of materials for the direct air capture of CO_2_ (DAC). Practical implementation of adsorption-based DAC requires adsorbents that can be used for thousands of adsorption–desorption cycles without significant degradation. We examined the potential degradation of adsorbents by a mechanism that appears to have not been considered previously, namely, ozonolysis by trace levels of ozone from ambient air. We focused on amine-appended metal–organic frameworks, specifically amine-functionalized Mg_2_(dobpdc), as a representative DAC adsorbent. Estimates based on the number of amine sites in these adsorbents and the ozone concentration in air suggest that degradation by ozone may be relevant over thousands of adsorption–desorption cycles if reactions with adsorbed ozone are fast. We used density functional theory calculations to estimate reaction rates for amine groups and carbon–carbon double bonds in amine-functionalized Mg_2_(dobpdc).

## Introduction

Direct air capture (DAC) of CO_2_ is a promising technology in future efforts for decarbonization. If DAC is implemented at large scales and along with CO_2_ sequestration then it can directly mitigate anthropogenic CO_2_ emissions.^1−4^ Potential advantages of DAC over other decarbonization strategies include flexibility in terms of location and scale of deployment and the ability to capture CO_2_ already emitted in the atmosphere. Large-scale implementation of DAC will require careful process optimization to reduce costs.^5^ An important strategy for reducing operating costs for DAC is to use capture materials that have long lifetimes under practical conditions. In DAC processes using porous adsorbents,^6−10^ it is likely to be desirable to use an adsorbent for tens of thousands of adsorption/regeneration cycles to achieve cycle times of ∼1 h and adsorbent lifetimes of >1 year.

When aiming to develop adsorbents that reach the long lifetimes described above, it is important to consider the full range of processes that could reversibly or irreversibly degrade the material’s performance. Degradation of adsorbents can occur by slow accumulation of trace components that are difficult to remove during regeneration.^11−17^ These effects can, in some cases, be reversed by more aggressive regeneration (e.g., heating to higher temperatures) at well-defined intervals. Degradation can also potentially occur via chemical reactions that irreversibly change the material’s structure. Corrosion of metals in ambient environments is a well-known example of this kind of degradation where the interaction of metals with oxygen, moisture, pollutants, or other corrosive agents in the surroundings leads to loss of functionality and structural disintegration. In this paper, we consider whether irreversible degradation due to a ubiquitous trace component of air, ozone, will be of concern for the long-term stability of porous adsorbents for DAC.

Ozone is present in ambient air at levels of ∼10 ppb and is an aggressive oxidant.^18−20^ Ozone plays an important role in many chemical reactions in the troposphere. When the ozone concentration is high due to emissions from burning fossil fuels it becomes a harmful pollutant and part of smog.^21,22^ Since the industrial revolution, ozone levels have risen significantly due to the burning of fuels.^23^ As is the case for CO_2_, there is considerable seasonal variation in ozone levels in air.^5^ In particular, more heat and sunlight in the summer create more ozone, leading to higher pollution levels during the summer months.^24^

It is known that long-term exposure to ozone at low concentrations (ppb levels) can degrade highly robust polymers such as vulcanized rubber.^25^ This observation raises the possibility that DAC adsorbents may also be susceptible to attack by ozone when they are used over thousands of capture/regeneration cycles. Because amine-functionalized adsorbents play an important role in direct air capture of CO_2,_^26−30^ we considered possible reactions between ozone and adsorbents of this type. Experiments and calculations have shown previously that ozone can react directly with alkyl amines and carbon–carbon double bonds in alkyl and aromatic alkenes.^31−39^ These reactions could potentially degrade the active amine sites available for the binding of CO_2_ in DAC-relevant adsorbents. The reaction of ozone with alkyl amines leads to the formation of products such as imines, aldehydes, nitrates, water, and O_2_, among other species.^31,33,37−39^ Furuhama et al. calculated reaction profiles for the reaction of ozone with methylamine (MA), dimethylamine (DMA), and trimethylamine (TMA) in the gas phase, and their results are consistent with earlier experimental observations.^32^ Their calculations showed that the initial reactions of amine (MA, DMA, or TMA) with O_3_ consist of (i) H-abstraction from methyl groups by O_3_, (ii) H-abstraction from an amino group by O_3_, and (iii) covalent N–O bond formation between an amino group and an O_3_ with H-abstraction from an amino group. They reported that the contribution of reaction (iii) appears to be small. Hence, we focused on modeling the H-abstraction pathways in our study. They found structures for prereaction complexes (amine···O_3_ noncovalent bond formation) and intermediates (amine··· O_3_ covalent bond formation) that are connected by transition states.^32^ The prereaction complex is a DFT-optimized geometry of the reactant state before the actual reaction proceeds to the intermediate product via a transition state. The products, after the first step, undergo further conversion to yield a wide variety of final products. In amine-functionalized MOFs, amine sites may be susceptible to attack by ozone, resulting in loss of CO_2_ capture sites and subsequent adsorbent degradation.

Ozone can react with carbon–carbon double bonds via the Criegee mechanisms (cyclo addition) involving a symmetric transition state with primary ozonide formation or a De More mechanism involving a biradical transition state with stepwise ozone addition. Systematic quantum chemistry calculations of ozone addition to various olefins show that in the gas phase, the Criegee mechanism dominates in most cases.^35,40−45^ Krisyuk et al.^45^ investigated the mechanism of initial ozonolysis of olefins and *trans*-1,3-butadiene using B3LYP DFT, a B2PLYP double hybrid method based on DFT and MP2, and a CCSD method with an aug-cc-pVDZ basis set. Because of the high exothermicity of the process, the resulting primary products undergo further conversion to yield a wide variety of final products such as aldehydes, CO_2_, CO, H_2_O, H_2_, etc. Considering initial ozone addition as a rate-determining step, Krisyuk et al. calculated rate constants using transition state theory.^45^ Chapleski et al. reported a theoretical study on the ozonolysis of fullerene.^43^ They characterized reactions of O_3_ with C_60_ that include both the formation of primary ozonide and subsequent dissociation reactions of this intermediate that lead to C–C bond cleavage that ultimately results in an open-cage structure. Subsequent reaction of the open-cage C_60_O_3_ product with a second ozone molecule opens a low-energy reaction pathway that results in cage degradation via the loss of a CO_2_ molecule.^43^ Reaction of ozone with benzene has been studied in the literature.^33,35,46^

As described above, ozone can react with amine groups and carbon–carbon double bonds in amine-functionalized Mg_2_(dobpdc), leading to structural distortions in the sorbent caused by the formation of various stable products, ultimately resulting in material degradation. Directly determining the effects of the ppb level of ozone on the performance of DAC adsorbents experimentally may be challenging, especially if the impacts of degradation accumulate slowly over thousands of capture/regeneration cycles. Because of the lack of information available on this topic, it is useful to use quantum chemistry calculations to provide insight into the potential impacts of ozone on adsorbents. To this end, a key aim of this paper is to examine the potential degradation of a representative amine-based adsorbent by ozone at the mechanistic level. Specifically, we study amine-functionalized Mg_2_(dobpdc), a metal–organic framework (MOF) that has been extensively studied for CO_2_ capture at conditions relevant to DAC.^29,47−50^ We study the initial steps of H-abstraction in the reaction of ozone with amine groups appended to open metal sites and ozone addition to the carbon–carbon double bond present in organic linkers to form POZ (primary ozonide). These initial reaction steps in ozonolysis may be rate-determining steps in overall degradation pathways, similar to the gas-phase studies highlighted above. To investigate these reactions at a mechanistic level, we employed density functional theory (DFT) calculations to quantify the reaction rates of ozone with the amine-functionalized Mg_2_(dobpdc).

## Computational Methods

**Figure 1:**
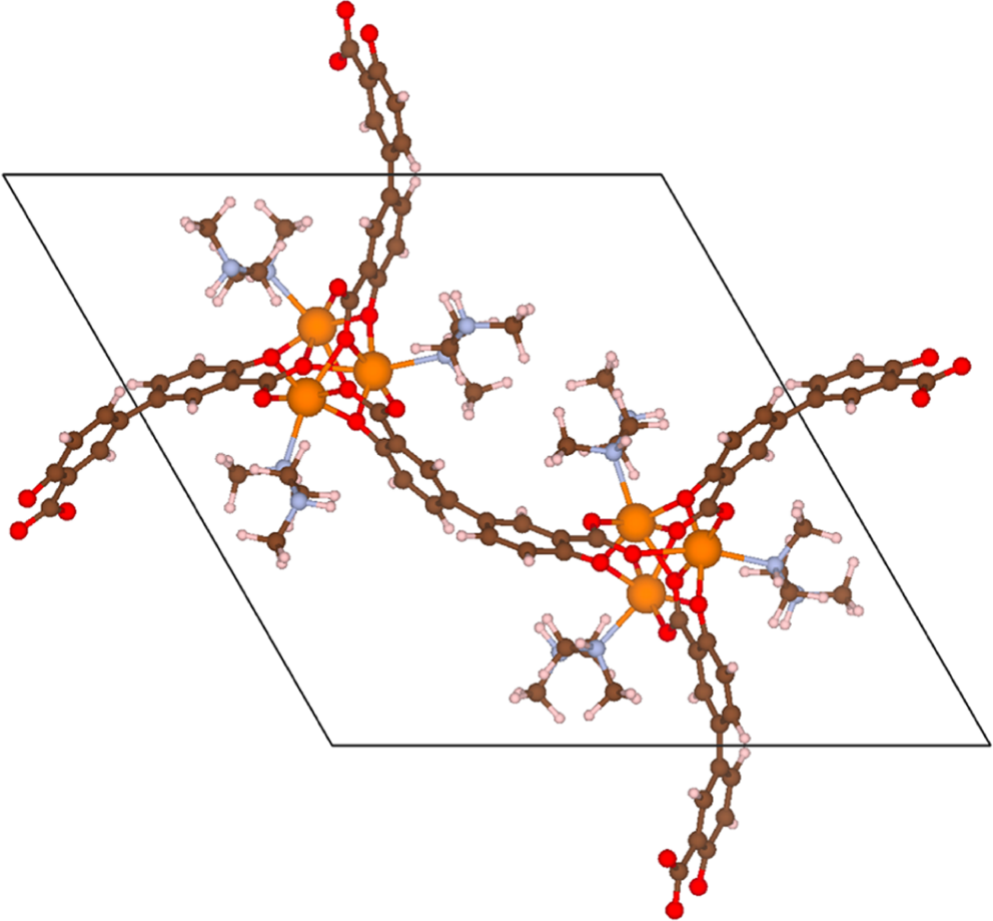
Unit cell of mmen-Mg_2_(dobpdc) depicting 6 *N*,*N*-Dimethylethylenediamine molecules appended to open metal sites in Mg_2_(dobpdc).

We focused on a MOF that is representative of a wide variety of amine-functionalized DAC adsorbents. Although crystalline materials such as MOFs may or may not be the specific adsorbents in practical settings for DAC, insights obtained from this work will be helpful for considering a wide class of DAC-relevant adsorbents. We selected Mg_2_(dobpdc) as the host framework for this work. Mg_2_(dobpdc) (dobpdc^4–^ = 4,4′-dioxidobiphenyl-3,3′-dicarboxylate), which has been studied extensively with density functional theory (DFT) calculations,^51−55^ is a MOF with a high density of open metal sites that can readily be functionalized. Diamine (*N*,*N*-Dimethylethylenediamine) appended Mg_2_(dobpdc) has been shown to have useful CO_2_ capture properties under the ultradilute conditions important for DAC.^29,47−50^ In this study, we considered *N*,*N*-Dimethylethylenediamine appended to open metal sites in Mg_2_(dobpdc). A single unit cell of Mg_2_(dobpdc) contains six open metal sites. If only one of these open metal sites is attached to *N*,*N*-Dimethylethylenediamine, we refer to this structure as 1-mmen-Mg_2_(dobpdc). However, if all six open metal sites are attached to *N*,*N*-Dimethylethylenediamine, the structure is referred to as mmen-Mg_2_(dobpdc). Figure 1 shows the structure of this material in a situation in which every open metal site has been functionalized with a diamine molecule.

We obtained the structure for mmen-Mg_2_(dobpdc) from the work by Lee et al.^52^ Diamine molecules coordinate to open metal sites in the MOF by forming a Mg-N bond. Using the mmen-Mg_2_(dobpdc) structure file and Mercury visualization software, we generated a 1-mmen-Mg_2_(dobpdc) structure where only one open metal site in the unit cell is appended with a diamine molecule. In order to make these structures computation-ready, full cell geometry optimization was performed with plane-wave DFT calculations using the Vienna Ab initio Simulation Package (VASP) with D3 dispersion corrections^56^ and the Perdew–Burke–Ernzerhof exchange–correlation (PBE) functional.^57^ Geometry optimization using a conjugate gradient method and an energy cutoff of 600 eV was performed with the relaxation of both lattice parameters and ionic positions until all ionic forces reached less than 0.01 eV/Å. A 1 × 1 × 1 *k*-point mesh was used for all calculations. Atomic charges for the optimized geometry were assigned by the DDEC6 method. DDEC partial charges accurately reproduce the electrostatic potential in the MOF pores and hence provide an accurate representation of electrostatic interactions between the MOF and adsorbates with polar and quadrupolar interactions.^58^ Optimized structure files for all of the structures studied in this work are included in the Supporting Information.

The climbing nudged elastic band (cNEB) method was used to quantify energy barriers and identify the transition state for the H-abstraction from amine groups by ozone and ozone addition to carbon–carbon double bonds.^59,60^ Transition state tools for VASP (VTST)^61^ were used for cNEB calculations. We used a force convergence criterion of 0.01 eV/Å for the cNEB calculations. For cNEB calculations, geometry optimization was performed for the prereaction complex and intermediate product, where the cell shape and cell volume were kept fixed, and the same functional and other computational details were used as those mentioned for the previous DFT calculations. We initially generated five images between the prereaction complex and intermediate products using interpolation and manually tuned the image coordinates in cases where interpolation resulted in overlapping atoms or chemically unplausible structures. Vibrational frequencies were estimated using finite-difference calculations with a step size of 0.01 Å, considering the movement of all atoms and keeping the cell volume and shape fixed. In our DFT calculations for transition state (TS) structures we observed more than one imaginary frequency in some cases; however, the largest imaginary frequency observed in all our calculations corresponds exclusively to the reaction step, specifically H-abstraction or ozone addition to the C=C bond, and the other imaginary frequencies are relatively small and do not associate with the reaction step. This can be confirmed through the frequency visualization files provided in the Supporting Information (SI) for all calculations. Vibrational frequencies of reactants, transition states (TS), and intermediate products were further used in free energy estimation. We denote by Δ*E* the DFT electronic energy difference between the TS and the prereaction complex and the heat of reaction Δ*H*_1_ as the DFT electronic energy difference between the intermediate product and the prereaction complex. Input files for all of the DFT calculations and POSCAR files for the prereaction complex, TS, and intermediate products are included in the SI.

Considering the initial reactions as the rate-determining step, we used the Eyring equation based on transition state theory (TST) to estimate rate constants using^32,43,45,62,63^
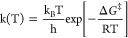
1where T is the reaction temperature (K), Δ*G*^‡^ is the Gibbs free energy of activation, k_B_ is the Boltzmann constant, and h is the Planck constant. The term  can be interpreted as the frequency with which reactant molecules in the prereaction complex attempt to cross the energy barrier to form products, and the Gibbs free energy of activation Δ*G*^‡^ represents the energy barrier that must be overcome for the reaction to proceed.^62,63^

Free energies were deduced assuming harmonic vibrations and the ideal gas limit as implemented in the ASE Thermochemistry package.^64^ The HarmonicThermo class in the thermochemistry package supports the calculation of internal energy, entropy, and free energy in the approximation that all degrees of freedom of adsorbates, i.e., 3*n*, where *n* is the number of atoms, are treated harmonically and it does not involve the translational and rotation degrees for freedom. This class returns the Helmholtz free energy; if the PV term (in H = U + PV) is neglected, this can also be interpreted as the Gibbs free energy. In our calculations, the HarmonicThermo class was used to estimate the free energies of all of the species except the ozone molecule. The free energy for the gas-phase ozone was estimated by using the IdealGasThermo class in the thermochemistry package. In the IdealGasThermo class, the thermodynamic quantities of ideal gases are calculated by assuming that all spatial degrees of freedom are independent and separable into translational, rotational, and vibrational degrees of freedom. Both of these classes use vibrational energies estimated using DFT calculation as described above. The Gibbs free energy of reaction (Δ*G*_1_) is defined as the difference in free energy between the intermediate product (IP) and reactants/prereaction complex. In all cases, free energies of intermediate products are estimated using the HarmonicThermo class.

For the ozonolysis reaction of isolated molecules like *N*,*N*-Dimethylethylenediamine and benzene, the Gibbs free energy of activation is defined as the free energy difference between the TS and the reactants, Δ*G*^‡^ = *G*_TS_ – *G*_mmen_ – *G*_O_3__ for *N*,*N*-Dimethylethylenediamine, and Δ*G*^‡^ = *G*_TS_ – *G*_Benzene_ – *G*_O_3__ for benzene. In both cases, the IdealGasThermo class was used to estimate the free energy for ozone, while the HarmonicThermo class was used to estimate the free energies of TS, mmen, and benzene. For the reaction of ozone with amine sites and carbon–carbon double bonds in MOFs, the Gibbs free energy of activation is taken to be the free energy difference between the TS and the prereaction complex, that is, the DFT-optimized configuration of ozone inside the MOF pores Δ*G*^‡^ = *G*_TS_ – *G*_PRC_. The HarmonicThermo class was used to compute the free energies for the TS and PRC in these cases.

The rate constant in eq 1 describes the reaction rate from the prereaction complex. When comparing with experimental data for bulk systems, it is often useful to express the net reaction rate *k*_g_(T) by treating the gas phase as an ideal gas, giving^65^
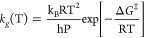
2We used *k*_g_(T) to compare our results for the ozonolysis of *N*,*N*-Dimethylethylenediamine and benzene with previous literature results for gas phase or bulk phase systems. For comparing reaction kinetics across different systems, such as the reactions of ozone in MOFs to the gas phase results of *N*,*N*-Dimethylethylenediamine and benzene, we solely used the rate constant k(T) provided by eq 1. Python scripts for the rate constant and free energy estimations are included in the SI.

To estimate overall ozonolysis rates in MOFs, it is necessary to estimate the adsorbed ozone concentration, C_O_3__. Because of the low concentration of ozone in ambient air and ozone’s relatively low molecular weight, the Henry constant for ozone in an adsorbent can be used to predict the adsorbed concentration of ozone. Henry’s constants are exponentially proportional to the heat of adsorption,^66^ with a temperature dependence given by , where ρ_*s*_ is the density of the adsorbent, and Δ*H*_ads_ is the heat of adsorption. Although extensive molecular simulations have been performed to predict Henry’s constants for the adsorption of many molecules in MOFs,^67^ there is currently no force field available for ozone that can be reliably used in molecular simulations for this purpose. However, the ratio of Henry’s constants for O_2_ and O_3_ can be estimated by . To estimate the heat of adsorption for ozone, based on the number of oxygen atoms in O_2_ and O_3_, the heat of adsorption for ozone can be approximated as follows: Δ*H*_ads,O_3__ ≈ 1.5Δ*H*_ads,O_2__. The Henry’s constant and the heat of adsorption for O_2_ were predicted with Widom insertion molecular simulations using the TraPPE force field^68^ for O_2_ with the RASPA code.^69^

## Results and Discussion

Before performing a detailed analysis of ozonolysis rates, it is useful to make an estimate of the possible impact of ozonolysis on a DAC adsorbent. For this purpose, we consider a plausible DAC process using an amine-functionalized adsorbent in which every ozone molecule entering the adsorbent bed reacts irreversibly with an amine site. This is of course a simplistic upper bound estimate on the potential impact of ozone, since ozone molecules could potentially exit the bed without entering the adsorbent’s pores or else enter a pore but not react before being desorbed. For specificity, we considered a DAC process model described by Sabatino et al.^70^ and assumed that mmen-Mg_2_(dobpdc) is used as the adsorbent in this process. The unit cell volume of this MOF (*V*_MOF_) is 2.5 × 10^–21^ cm^3^ and the MOF’s crystalline density (ρ_MOF_) is 988 kg/m^3^. A unit cell of mmen-Mg_2_(dobpdc) has 6 diamine molecules and thus the concentration of amine sites is 2.4 × 10^24^ molecules/kg. For the process described by Sabatino et al.,^70^ reported productivity ranges from 3.8 to 10.6 kg·CO__2__·m^–3^·h^–1^, the adsorption cycle time ranges from 100 to 10,000 s, and the air flow rate varies from 0.28 to 5.09 . For the upper bound estimations, we considered a productivity of 7.2 kg·CO__2__·m^–3^·h^–1^, an adsorption time of 1 h, and an air flow rate of 2.5 . Using a bulk ozone concentration in the air of 10 ppb gives the number of ozone molecules passing over the adsorbent bed in one cycle as 2.4 × 10^21^ molecules. For the purposes of this upper bound estimate, we assume that all of these ozone molecules react with amine sites in the MOF. In a DAC process modeled by Sabatino et al., which uses a structured contactor based on a commercial approach, the volume of the contactor in a single bed is 3.375 m^3^, which corresponds to 552 mol of CO_2_ captured in one cycle. Amine efficiency, defined as the amount of CO_2_ adsorbed divided by the amount of amine present in the material, is a useful way to quantify the effectiveness of amine-based sorbents. The highest possible chemisorptive amine efficiency of a supported amine material is determined by the CO_2_–amine interaction chemistry. If two amines are necessary to capture one CO_2_ molecule by forming ammonium carbamate pairs when water is not present, the maximum theoretical amine efficiency is 50%.^71^ Studies of CO_2_ capture under DAC-relevant conditions have reported amine efficiencies of 5–88%.^72−75^ We therefore assumed a 25% efficiency of CO_2_ capture for practical cyclic adsorption. Thus, to capture the 552 mol of CO_2_ the number of amine sites required in the adsorbent bed would be 1.33 × 10^27^. Assuming that each ozone molecule degrades one amine site implies a net amine degradation rate of 0.0002% per cycle. This degradation rate would be insignificant over the course of typical laboratory experiments, but if the bed was used to capture 1000 tons of CO_2_ over 40,000 cycles it would lead to cumulative degradation of approximately 8%.

The description above is, of course, quite schematic, but it suggests that there may be situations where degradation of DAC adsorbents by ozone may play a relevant role in practical situations. Degradation can also occur concomitantly due to other contaminants and oxidative processes. Once degradation begins from ozone or other sources, it is possible that cascading effects from increased reactivity of degraded sites could accelerate the overall degradation rate. This qualitative analysis of ozonolysis motivated us to examine the degradation caused by ozone at a mechanistic level, aiming to gain more accurate insights into reaction rates for ozonolysis in amine-functionalized MOFs.

### Reaction of *N*,*N*-Dimethylethylenediamine with O_3_

Furahama^32^ et al., in their theoretical study of reactions of amines with ozone, used high-level quantum chemistry methods such as UB3LYP, UM062X, UCCSD, and UCCSD(T) to incorporate the biradical character of ozone. These high-level calculations are possible for the ozonolysis of the small molecules in the gas phase, but it is impractical to perform similar high-level calculations for a large extended solid such as a MOF. Comparison between results with high-level methods and more computationally efficient B3LYP DFT calculations for gas-phase species showed that the inexpensive DFT method provides results that are qualitatively correct.^44,45^ Because it would be advantageous to perform plane-wave DFT calculations for the periodic solids of interest to us, it would be very useful if nonhybrid functionals could be used in DFT calculations. We therefore first benchmarked the accuracy of DFT calculations with PBE functional in VASP to literature results^32^ for gas-phase reactions of dimethylamine with ozone.

**Figure 2:**
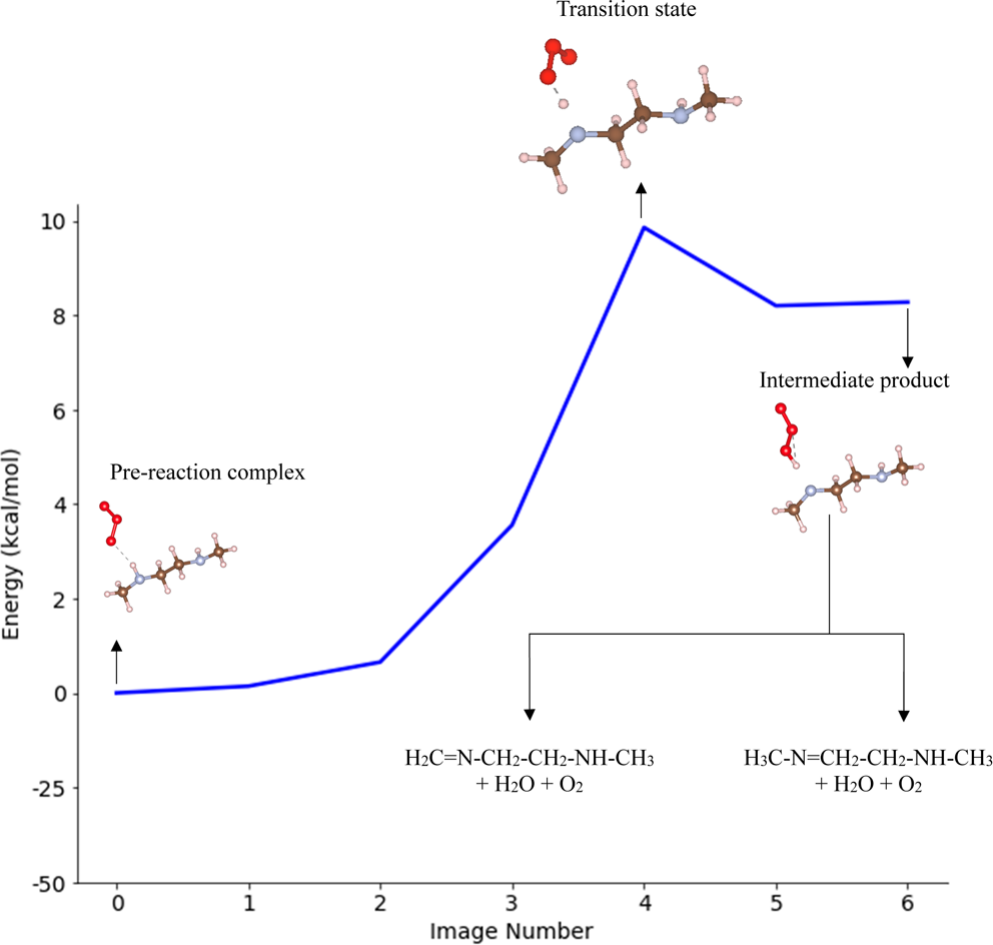
Energy diagram of a two-step reaction pathway for the reaction of *N*,*N*-Dimethylethylenediamine (mmen) and ozone (O_3_). The prereaction complex (Image 0) and the intermediate product (Image 6) are connected through the transition state (TS) (Image 4). Relative energy values (kcal/mol) were determined by using DFT calculations with the PBE functional.

We performed a series of calculations for the gas-phase reaction between *N*,*N*-Dimethylethylenediamine and ozone. Furuhama et al. showed that the reaction could proceed through H-abstraction from either the amine group or the methyl group.^32^ We generated two plausible cases for the intermediate product. In case 1, the hydrogen is abstracted from the amino group, resulting in the intermediate product shown in Figure 2. We performed geometry optimization for both the prereaction complex and the intermediate product. In the subsequent step, the O_3_---H species could further abstract another hydrogen from the alkyl group, leading to the formation of stable products such as imine, water, and O_2_, as depicted in Figure 2. Tuazon et al., in their experimental studies on the reaction of dimethylamine with O_3_, reported the formation of stable products, including H_3_CN=CH_2_, HCHO, HCOOH, CH_3_NO_2_, CO_2_, and CH_3_NHCHO.^31^ In case 2, the hydrogen is abstracted from the methyl group. However, the intermediate product after geometry optimization was H_3_C–NH–CH–OH–NH–CH_3_ (see Figure S1). Since the formation of this species was not observed in the experimental study by Tuazon et al., we did not investigate this mechanism further. We performed climbing nudged elastic band (cNEB) calculations as described above to quantify energy barriers and identify the transition state for H-abstraction from the amine group by ozone.

Furuhama et al., in their study of H-abstraction from the amine group in the dimethylamine molecule using the UCCSD(T)/aug-cc-pVDZ level of theory, estimated an electronic energy difference Δ*E* between the TS and prereaction complex of 8 kcal/mol. We used *N*,*N*-Dimethylethylenediamine (mmen) as a secondary amine molecule because of its relevance to our later calculations. Our PBE DFT calculations estimated an electronic energy difference Δ*E* between the TS and prereaction complex of 10 kcal/mol for H-abstraction from the amine group, consistent with Furuhama et al’s prediction. The estimated heat of reaction (Δ*H*_1_) for hydrogen abstraction is 8 kcal/mol, indicating that the H-abstraction step is endothermic. We estimated the rate constant for this reaction using the methods described above. Eq 1 estimated a rate constant of 1.8 × 10^3^ s^–1^, while the gas-phase reaction rate from eq 2 is 7.4 × 10^–17^ cm^3^·molecule^–1^·s^–1^. This gas-phase reaction rate constant is in reasonable agreement with literature results for a similar secondary amine molecule, dimethylamine. Specifically, this includes the computed rate constant of 3.2 × 10^–18^ cm^3^·molecule^–1^·s^–1^ by Furuhama et al.^32^ from calculations at the UCCSD(T)/UM062X/aug-cc-pVDZ level and the experimental estimate of 1.67 × 10^–18^ cm^3^·molecule^–1^·s^–1^ by Tuazon et al.^31^ Moreover, Tuazon et al. report an ozone decay rate in the range of 0.2 × 10^3^ s^–1^ to 1.6 × 10^3^ s^–1^, and eq 1 estimated a rate constant of 1.8 × 10^3^ s^–1^, in agreement with the reported results.

This comparison suggests that performing DFT calculations with the PBE functional can give qualitatively accurate results for these reactions while still allowing calculations to be performed for amine groups inside the periodic crystals.

### Reaction of 1-mmen-Mg_2_(dobpdc) with O_3_

**Figure 3:**
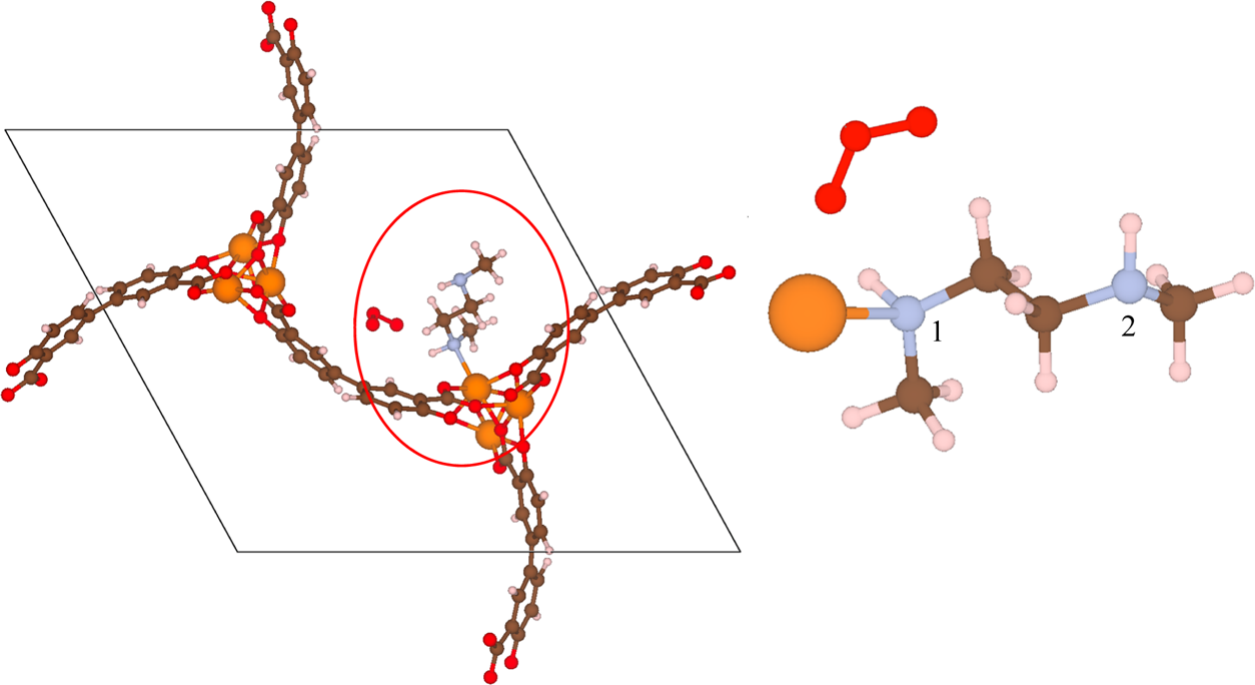
Unit cell of 1-mmen-Mg_2_(dobpdc) depicting only one *N*,*N*-Dimethylethylenediamine molecules appended to the open metal site in Mg_2_(dobpdc). The red oval highlights an adsorbed group of an adsorbed O_3_ molecule.

We used PBE DFT calculations to examine the reaction of ozone with *N*,*N*-Dimethylethylenediamine attached to an open metal site in Mg_2_(dobpdc) (see Figure 3). We included the effects of dispersion using D3 corrections, although we anticipate that the effect of dispersion on the reaction energetics is small.

We considered two cases: in case 1, ozone abstracts hydrogen from the nitrogen attached to a Mg site in the MOF framework, while in case 2, ozone abstracts hydrogen from a nitrogen that is farther from Mg. In both cases, a similar reaction pathway, as shown in Figure 2, was observed, with the reaction proceeding through the formation of O_3_---H as an intermediate product. In the subsequent steps, the O_3_---H species could further abstract another hydrogen from the alkyl group, resulting in the formation of imines. The estimated Δ*E* is 25 kcal/mol in case 1 and 12 kcal/mol in case 2. The resulting Gibbs free energy of activation for H-abstraction in case 1 is 17 and 6 kcal/mol in case 2. This shows that the situation in case 2 is similar to the results for gas-phase *N*,*N*-Dimethylethylenediamine, while in case 1, the close proximity of the MOF framework has a strong influence on the activation energy. This description also applies to the thermochemistry of the reactions. The computed heat of reaction (Δ*H*_1_) for hydrogen abstraction is 16 kcal/mol in case 1 and 7 kcal/mol in case 2 (see Figure S2). The heat of the reaction in case 2 is comparable to that of isolated N,N-Dimethylethylenediamine. In all of the ozonolysis reactions studied in this paper, the initial reaction step is an elementary step where one ozone molecule reacts with one amine site to abstract hydrogen. Thus, we can quantify the reaction speed and degradation of amine sites by using the relative comparison of reaction rate constants. We used eq 1 to estimate the rate constant for the reaction at 1 atm and 298.15 K for both cases. Due to the higher activation energy barrier for H-abstraction, the estimated rate constant of 3.2 s^–1^ in case 1 is significantly lower than that of case 2, where the estimated rate constant is 2.6 × 10^8^ s^–1^. This outcome suggests that the reaction proceeds much faster in the MOF than for *N*,*N*-Dimethylethylenediamine in the gas phase. Table 1 summarizes the reaction energetics and rates for these and other ozonolysis reactions that we have considered.

**Table 1:** Summarized Results of Ozonolysis Reactions Considered in This Study

reaction system	intermediate product	Gibbs free activation energy Δ*G*^‡^ (kcal/mol)	Gibbs free energy of reaction Δ*G*_1_ (kcal/mol)	rate constant, *k* (s^–1^)
MMEN + O_3_ (gas phase)	imine	13	14	1.8 × 10^3^
MOF (1 mmen) + O_3_	case 1—imine	17	12	3.2
	case 2—imine	6	8	2.6 × 10^8^
MOF (6 mmen) + O_3_	case 1—imine			
	case 2—imine	17	10	1.08
MMEN + O_3_ + H_2_O	imine	16	16	1.3 × 10^1^
C_6_H_6_ + O_3_	POZ	26	5	3 × 10^–7^
C=C bond in mmen-Mg_2_(dobpdc) + O_3_	POZ	14	–7	1.7 × 10^2^

### Reaction of mmen-Mg_2_(dobpdc) with O_3_

In the previous section, we observed the effect of a Mg metal site and a MOF environment on reaction energetics. We next considered the case where all of the six open metal sites in the unit cell of Mg_2_(dobpdc) are appended with N,N-Dimethylethylenediamine (see Figure 1). As mentioned above, we considered ozone abstracting hydrogen from the nitrogen attached to the magnesium metal site (case 1) and ozone abstracting hydrogen from nitrogen that is farther from magnesium (case 2). Because of the higher density of diamine molecules, our cNEB calculations did not converge successfully for case 1, so it was not explored further. In Case 2, the estimated Δ*E* is 20 kcal/mol, while the Gibbs free energy of activation Δ*G*^‡^ for H-abstraction is 17 kcal/mol. Figure S3 shows the reaction energy diagram for case 2. The energy barrier in this scenario is higher than in the previous Case 2 of 1-mmen-Mg_2_(dobpdc) scenario, presumably due to the crowding effect of the multiple amine molecules in the MOF’s pore. For this reaction, the estimated heat of reaction Δ*H*_1_ is 15 kcal/mol, and the rate constant from eq 1 is 1.08 s^–1^. These results suggest that one possible consequence of having very high diamine loadings in the MOF may be slower ozonolysis reactions for specific amine sites.

### Ozonolysis of *N*,*N*-Dimethylethylenediamine with O_3_ in the Presence of Water

It is well-known that the presence of water affects the reaction energetics and speciation of CO_2_ coordinating with amine sites.^76−79^ Furthermore, adsorbed water is likely to be ubiquitous in performing DAC with amine-based adsorbents.^80^ It is therefore useful to consider whether the presence of coadsorbed water might affect ozonolysis rates. For simplicity, we approached this issue using calculations with gas-phase *N*,*N*-Dimethylethylenediamine rather than the more complex calculations that would be required for the amine-functionalized MOF. We used DFT calculations analogous to those described above to study this system. Specifically, we considered two water molecules around one of the amine sites in *N*,*N*-Dimethylethylenediamine so that we could capture H-bonding effects among water molecules, the amine site, and ozone. We performed geometry optimization for this system in the presence of ozone to obtain the optimized prereaction complex. As shown in Figure S4, the Δ*E* for the H-abstraction from the amine group in the presence of water increased to 15 kcal/mol as compared to that of 10 kcal/mol in the case of an isolated *N*,*N*-Dimethylethylenediamine. This increase in activation energy for hydrogen abstraction by ozone can be attributed to the changes due to the hydrogen bond between the amine group and the water molecule. The calculated Gibbs free activation energy at 298.15 K for the molecule in the presence of water (16 kcal/mol) is higher than that for the isolated molecule (13 kcal/mol). For this specific case, the predicted reaction rate for the molecule in the presence of water is lower than that of the isolated molecule.

### Reaction of Benzene with O_3_

**Figure 4:**
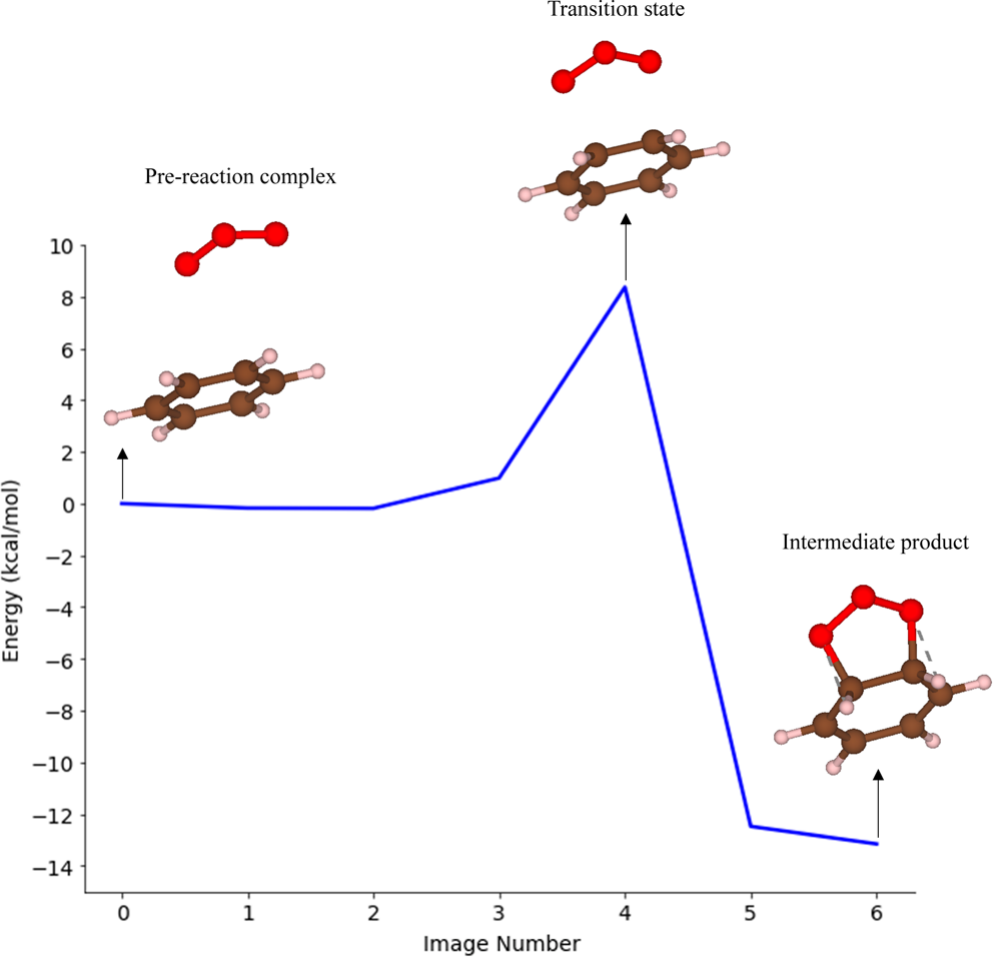
Energy diagram for the reaction of benzene and ozone (O_3_). The prereaction complex (Image 0) and the intermediate product (Image 6) are connected through the transition state (TS) (Image 4). Relative energy values (kcal/mol) were determined by using PBE DFT calculations.

The results above have focused on the ozonolysis of amine groups. MOFs such as Mg_2_(dobpdc) also contain other sites that can potentially be attacked by ozone, notably C=C double bonds. We also used DFT calculations to explore the potential reactivity of these bonds due to adsorbed ozone in the MOF. We first benchmarked our calculations against higher-level calculations from previous work for the gas-phase ozonolysis of benzene. A study on the reaction of O_3_ with benzene showed that B3LYP DFT calculations were consistent with reaction energies and barriers predicted by CCSD(T).^35^ We considered the addition of ozone to carbon–carbon double bonds by the Criegee mechanism with the formation of primary ozonide as an intermediate product. Figure 4 shows the reaction energy diagram for the primary ozonide formation from benzene from our PBE DFT calculations. Our DFT calculations gave Δ*E* of 8 kcal/mol, in reasonable agreement with the value of 10.7 kcal/mol at the B3LYP level by Hendricks et al.^35^ From this comparison, we concluded that the use of DFT with the PBE functional was acceptable. The PBE DFT heat of reaction Δ*H*_1_ in Figure 4 is −13 kcal/mol, indicating an exothermic reaction. At 298.15 K and 1 atm, the Gibbs free energy of activation is 26 kcal/mol, and the Gibbs free energy of reaction Δ*G*_1_ is 5 kcal/mol. The estimated rate constant for this process from our calculations is 3 × 10^–7^ s^–1^ (using eq 1), with a net reaction rate of 1.2 × 10^–26^ cm^3^·molecule^–1^·s^–1^ (using eq 2). A comprehensive review paper on the kinetics and mechanism of the gas-phase reactions of ozone with organic compounds under atmospheric conditions by Atkinson et al. reports a rate of 7 × 10^–23^ cm^3^·molecule^–1^·s^–1^ for the reaction of benzene with ozone and a decay rate of toluene, an aromatic molecule similar to benzene, of less than 1.7 × 10^–7^ s^–1^.^33^ Thus, our estimations are in reasonable agreement with the experimental observations. When benzene reacts with ozone, we assume that ozone behaves as an ideal gas, accounting for all degrees of freedom. The contributions of entropic energy from translational and rotational motions are significant in the calculation of free energies, leading to a higher Gibbs free energy barrier for the reaction at 298.15 K. The rate constant estimates derived from these energy barriers align well with the existing literature.

### Reaction of Carbon–Carbon Double Bond in mmen-Mg_2_(dobpdc) with O_3_

We performed DFT calculations analogous to those described above to describe the reaction of a carbon–carbon double bond in mmen-Mg_2_(dobpdc) with ozone (see Figure S5). In this material, there are three types of carbon–carbon bonds (see Figure S6): one type in which the carbon is part of a bridge between two aromatic rings in the linker, one in which one of the carbon is part of a bridge between a metal node and an aromatic ring, and a third in which both carbons have a hydrogen atom bonded to them and are not part of any bridge bond between another aromatic ring or metal node. We assumed that ozone addition via the Criegee mechanism at the first two types of sites is likely to be energetically unfavorable as it would result in considerable structural distortion. The third kind of carbon–carbon double bonds are similar to those in ozone addition to benzene and alkenes, so we considered these environments for our calculations.

The estimated Δ*E* of 11 kcal/mol for the reaction of ozone with a carbon–carbon double bond in the MOF is comparable to that of an isolated benzene molecule (see Figures 4 and S5). However, the computed Gibbs free energy barrier Δ*G*^‡^ for ozone addition in the MOF is 14 kcal/mol, which is significantly lower than that for a benzene molecule. This difference arises, at least in part, because of the differences in calculation methods for the two situations that were described in the Computational Methods section. The computed heat of reaction Δ*H*_1_ for ozone addition in the MOF is −9 kcal/mol, slightly higher than the Δ*H*_1_ of −13 kcal/mol for an isolated benzene molecule, and the estimated rate constant in this case is 1.7 × 10^2^ s^–1^. The main qualitative conclusion from this analysis is that once the prereaction complex is formed in the MOF, the ozonolysis reaction at ambient temperature is predicted to be fast.

### Ozonolysis Rates

The calculations described above give mechanistic insight into ozonolysis reactions for DAC adsorbents; however, to predict the net rate of ozonolysis, we need information about the concentration of ozone in the adsorbent pores. Here, we briefly discuss a method for estimating the adsorbed ozone concentration at equilibrium inside the MOF pores. By using molecular simulations for oxygen inside the MOF, as described in the Computational Methods section, we estimated the Henry’s constant for ozone. The heat of adsorption from molecular simulations is Δ*H*_ads,O_2__ = −12.7 kJ/mol for oxygen, giving an estimated heat of adsorption of Δ*H*_ads,O_3__ = −19.1 kJ/mol for ozone. The resulting Henry’s constant for oxygen is k_H,O_2__ = 1.9 × 10^–6^ mol·kg^–1^·Pa^–1^ and for ozone is k_H,O_3__ = 13.2 × 10^–6^ mol·kg^–1^·Pa^–1^. We can estimate the concentration of ozone in ambient air as 10 ppb, which corresponds to a partial pressure of P_O_3__ = 0.0010 Pa. Above, we discussed the upper bound estimates on ozone reactions in a particular adsorbent, estimating that capturing 552 mol of CO_2_ would require 1.33 × 10^27^ amine sites in the adsorbent bed. This number of amine sites corresponds to an adsorbent (mmen-Mg_2_(dobpdc)) mass of 554 kg. Using this information, the estimated number of adsorbed ozone molecules when this bed is at equilibrium with air at room temperature is N_O_3_,adsorbed_ = (k_H,O_3__) × (Mass of adsorbent) × P_O_3__ × 48 × 6.022 × 10^23^ = 2.14 × 10^20^.

N_O_3_,adsorbed_ provides just an estimate of the adsorbed ozone concentration at equilibrium inside the MOF pores. However, in a real process, ozone molecules will continuously react with reaction sites as they enter the system, making it challenging to predict the net ozone concentration inside the MOF pores at any given time. Therefore, to develop a more nuanced description of reactive ozone adsorption in future work it would be necessary to develop a process model that incorporates mass transfer resistances and the calculated reaction rate constants, integrating real-time ozone concentration data within the bed to predict the net ozonolysis reaction rates.

## Conclusions

Amine-functionalized adsorbents can play an important role in direct air capture (DAC) of CO_2_. Because practical applications of adsorbents for DAC will require adsorbent stability over a large number of adsorption cycles, there is a need to understand a range of processes that might contribute to the slow degradation of these materials. In this paper, we performed the first assessment of the potential effects of ozone from ambient air. Even though ozone is present at only trace levels in air, prior studies of molecular species have shown that ozone can be an aggressive oxidant for amine sites and carbon–carbon double bonds. In this study, we have investigated similar reactions in amine-functionalized Mg_2_(dobpdc), a representative DAC adsorbent. Upper bound estimates of the degradation of amine sites in this adsorbent in a representative DAC process model suggested that significant net degradation by ozone may be possible over large numbers of cycles. This estimate motivated us to use quantum chemistry calculations to examine the specific reaction mechanisms and rates for ozonolysis of mmen-Mg_2_(dobpdc).

We first benchmarked the accuracy of a computational approach that is feasible for periodic solids, DFT calculations at the PBE level, by estimating activation energies and rate constants for the reactions of ozone with small molecules such as N,N-Dimethylethylenediamine and benzene. Our results using this approach are in reasonable agreement with literature results using higher-level quantum chemistry methods. We then extended our calculations to study the initial steps in the reaction of ozone with amine groups appended to open metal sites (OMS) and ozone addition to the carbon–carbon double bond present in organic linkers.

We performed calculations for the 1-mmen-Mg_2_(dobpdc) system, where *N*,*N*-Dimethylethylenediamine is attached to one of the open metal sites (OMS) in a Mg_2_(dobpdc) unit cell. The computed Gibbs free energy barrier for H-abstraction by ozone at the amine site attached to the OMS is higher than for the gas-phase reaction of *N*,*N*-Dimethylethylenediamine, but the free energy barrier at an amine site far from the OMS is lower than for the gas-phase molecule. We also examined the effect of high levels of diamine functionalization in the MOF on reaction energetics. The computed energy barrier for H-abstraction in the presence of high diamine loading was higher than for the gas-phase reaction of the diamine molecule. These results indicate that the reactivity of amine sites inside the MOF varies depending on crowding effects and proximity to the MOF’s open metal sites, but there is likely to be a significant population of amine sites at which ozonolysis would be rapid once ozone is adsorbed in the pores. Ozone can also potentially attack carbon–carbon double bonds in the MOF. Using the same computational approach, we estimated the free energy barrier for primary ozonide formation via ozone addition to carbon–carbon double bonds in the organic linker of mmen-Mg_2_(dobpdc). Similar to the results for reactions with amine sites, our calculations indicate that ozonide formation with carbon–carbon double bonds could occur rapidly at ambient temperatures once ozone is present inside the MOF.

A thorough understanding of reactions with ozone during realistic DAC operations must consider not just the reactions of ozone in an empty adsorbent but the same reactions under conditions where the adsorbent contains significant amounts of CO_2_ and/or H_2_O. These coadsorbed species are unlikely to substantially change the reactivity to ozone of carbon–carbon double bonds since these bonds will not interact strongly with CO_2_ or H_2_O. To partially explore the impact for amine groups, we considered the ozonolysis of a gas-phase diamine molecule in the presence of water molecules. Perhaps surprisingly, our results did not indicate a large change in the reaction energetics associated with water coordinated to the diamine.

In this work, we independently studied two specific types of reactions in amine-functionalized MOF. In reality, these reactions, along with other reaction pathways associated with newly formed reactive intermediates, would occur simultaneously, resulting in an accelerated cumulative degradation rate compared to individual reaction rates. In future work, more detailed mechanistic studies exploring various reaction pathways after the initial rate-determining step would be useful to understand the cumulative effects of degradation in these materials.

There are several limitations to the results that we have presented. We have only considered a specific amine-functionalized MOF. This material was chosen because it has the characteristics of many amine-based adsorbents for DAC, but it would, of course, be interesting to know how generalizable our conclusions are to other materials. We have considered only the initial steps of ozonolysis, assuming that these steps are the rate-limiting processes in the degradation of the adsorbent. This assumption appears plausible based on earlier detailed studies of gas-phase molecules. Examples are known in other degradation processes for MOFs where initial degradation reactions can accelerate subsequent degradation in the vicinity of the original reaction.^81^ Our calculations cannot provide any insight into whether this phenomenon could occur for the ozone-related reactions we have studied. Our discussion implicitly assumes that these ozonolysis reactions cause irreversible degradation of the adsorbent that diminishes its capacity for CO_2_ capture, but we have not directly tested this idea. All of these issues would be interesting topics for further studies, but given that no information is previously available about the reactivity of MOFs to ozone, we feel that our results are useful even with these caveats. It would, of course, be interesting to test the stability of DAC adsorbents to ozone exposure experimentally. Because the estimates we have provided suggest that substantial degradation due to ozone in ambient air may only become apparent after thousands of adsorption cycles, it would be sensible to perform initial experiments using much higher concentrations of ozone.

A full process model incorporating the potential impacts of ozone on a DAC adsorbent is not currently feasible, but a validated model of this kind could be very useful in the development of accelerated testing protocols. In addition to the effects of coadsorption on reactivity mentioned above, a model of this kind would need to incorporate the temperature profile associated with CO_2_ desorption. CO_2_ desorption is typically achieved in DAC processes by heating the adsorbent. This heating will also remove adsorbed ozone molecules from the adsorbent but simultaneously greatly increase the reaction rates associated with the degradation reactions we have described. The properties of ambient air relevant for DAC operations, including the CO_2_ concentration, temperature, pressure, and humidity, have been shown to have strong variations on hourly and seasonal time scales and among different physical locations.^5,82^ Detailed treatments of the impacts of ozone on the long-term operation of DAC facilities would likely require real-world data on the spatial and temporal variation of ozone concentrations in air.

## Supporting Information Available

Reaction energy diagram for H-abstraction for Case 1 and Case 2 in 1-mmen-Mg_2_(dobpdc); energy diagram for the reaction of *N*,*N*-Dimethylethylenediamine (mmen) and ozone (O_3_) in the presence of water; energy diagram for the ozone (O_3_) addition to carbon–carbon bond in mmen-Mg_2_ (dobpdc); and unit cell of mmen-Mg_2_ (dobpdc) depicting three types of carbon–carbon double bonds in linker for possible ozone addition (PDF).

## Notes

Notice of Copyright: This work was authored by UT-Battelle, LLC, under contract DE-AC05-00OR22725 with the US Department of Energy (DOE). The publisher acknowledges the US government license to provide public access under the DOE Public Access Plan (http://energy.gov/downloads/doe-public-access-plan).
