# Supplementary material for: Assessment of Long-Term Degradation of Adsorbents for Direct Air Capture by Ozonolysis
Source: J Phys Chem C Nanomater Interfaces. 2024 Dec 20;129(1):899–909. doi: 10.1021/acs.jpcc.4c07054 (PMC11726669; doi:10.1021/acs.jpcc.4c07054)
Supplement: Supplementary file 1 — jp4c07054_si_001.pdf [file jp4c07054_si_001.pdf]

**Supplementary Information**

**Assessment of Long-term Degradation of Adsorbents for Direct Air Capture by Ozonolysis**

*Shubham Jamdade[1], Xuqing Cai[1] and David S. Sholl[2]*

[1]School of Chemical & Biomolecular Engineering, Georgia Institute of Technology, Atlanta, Georgia 30332-0100, United States
[2]Oak Ridge National Laboratory, Oak Ridge, TN 37830, United States

The ZIP file with DFT input files for all the geometry optimization and cNEB calculations described in this study is available at https://github.com/shubhamjamdade/JPC-Ozonolysis-DAC-Adsorbent-Supplementary-Information. Additionally, MOF CIF files and python scripts utilizing thermochemistry packages have been included in the ZIP file. Electronic energies and Gibbs free energies data for all the cases can be found in XLSX file.

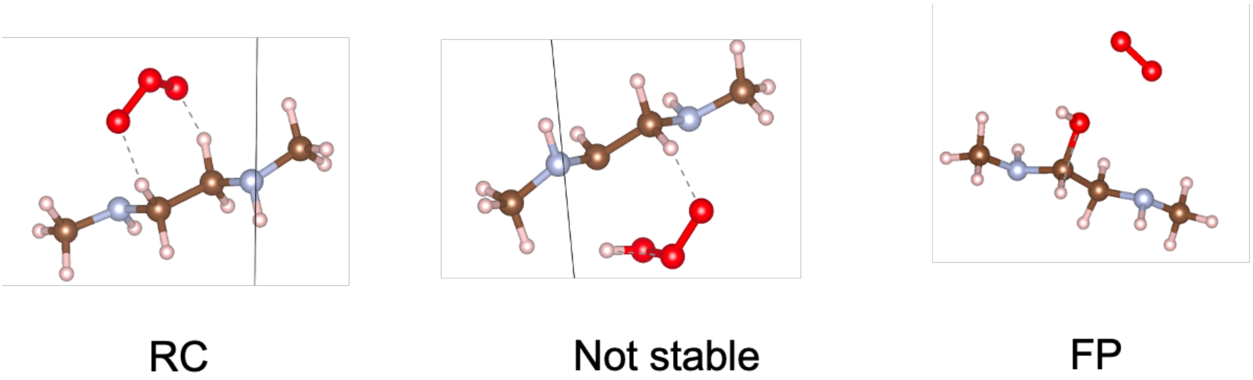


Figure S1: Reaction pathway for the reaction of N,N-Dimethylethylenediamine (mmen) and ozone ($O_3$) when hydrogen is abstracted from alkyl group

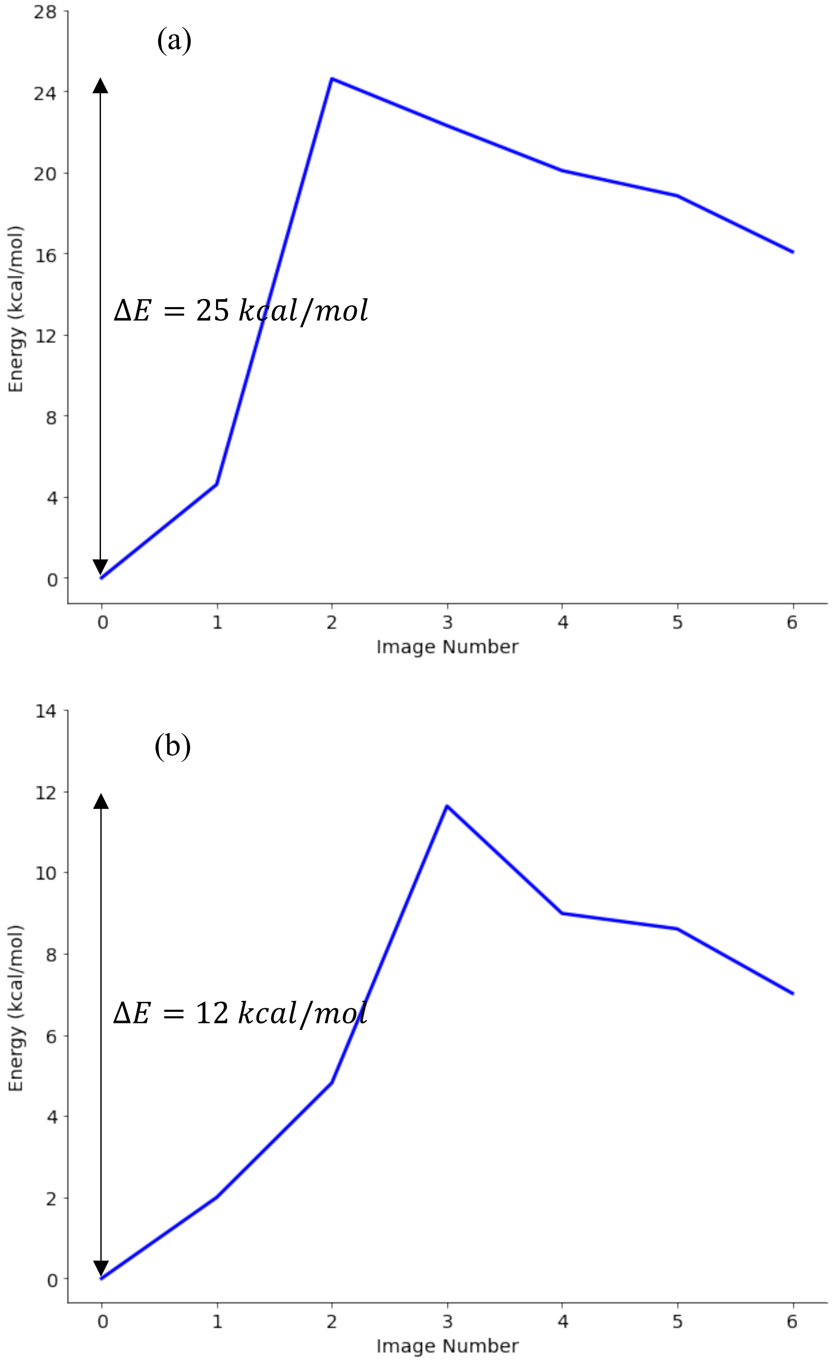


Figure S2: Reaction energy diagram for H-abstraction for (a) Case 1 (b) Case 2 in 1-mmen-$Mg_2$(dobpdc). The pre-reaction complex (Image 0) and the intermediate product (Image 6) are connected through the transition state, Image 2 in case 1 and Image 3 in case 2. Relative energy values (kcal/mol) were determined using DFT calculations. $\Delta E$ is the DFT electronic energy difference between transition state and pre-reaction complex.

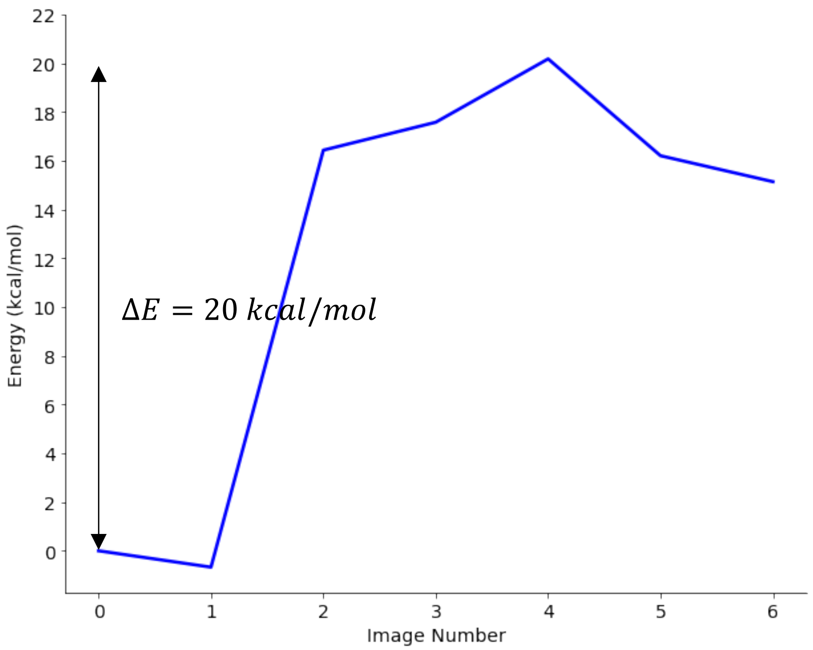


Figure S3: Reaction energy diagram for H-abstraction for Case 2 in mmen-$Mg_2$(dobpdc). The pre-reaction complex (Image 0) and the intermediate product (Image 6) are connected through the transition state, Image 4. Relative energy values (kcal/mol) were determined using DFT calculations. $\Delta E$ is the DFT electronic energy difference between transition state and pre-reaction complex.

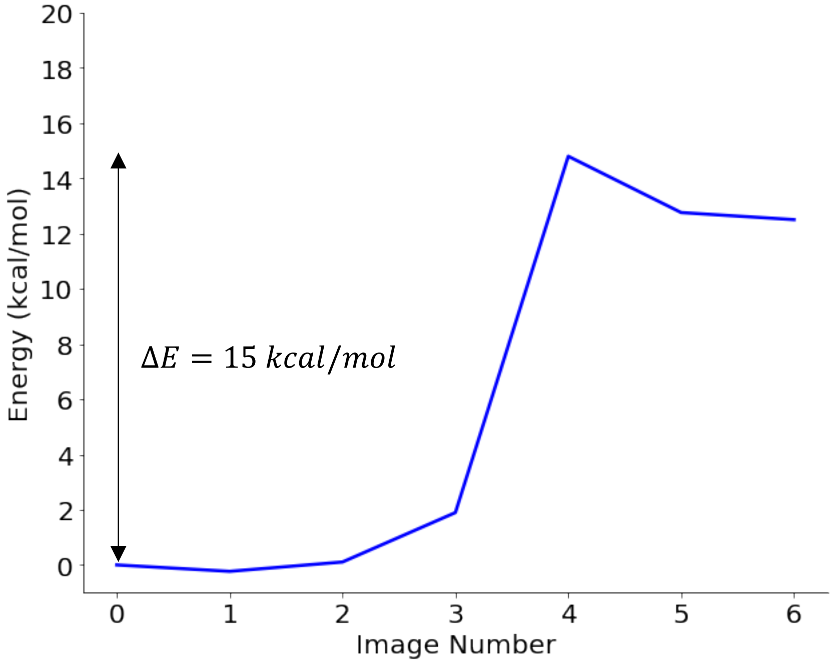


Figure S4: Energy diagram for the reaction of N,N-Dimethylethylenediamine (mmen) and ozone ($O_3$) in the presence of water. The pre-reaction complex (Image 0) and the intermediate product (Image 6) are connected through the transition state, Image 4. Relative energy values (kcal/mol) were determined using DFT calculations. $\Delta E$ is the DFT electronic energy difference between transition state and reactants.

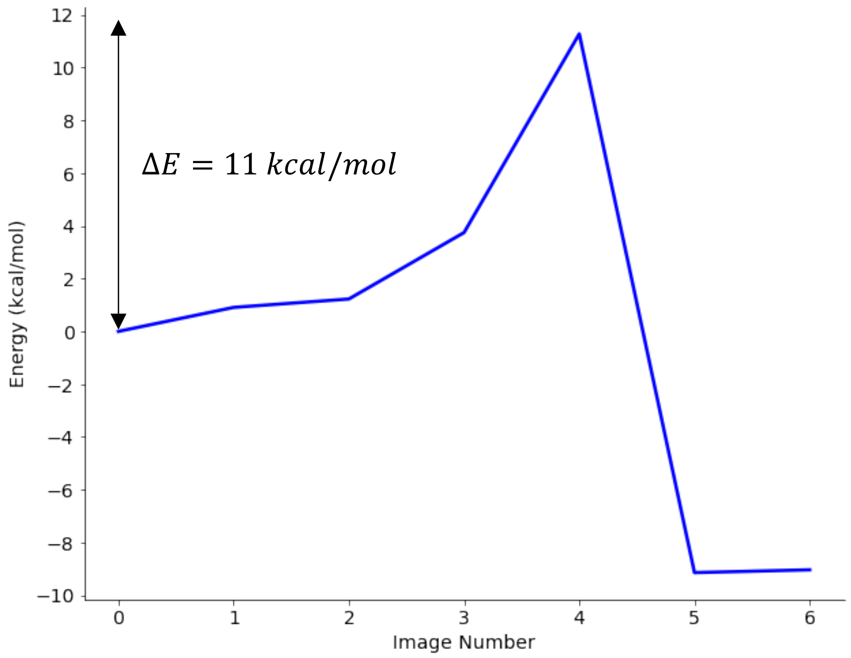


Figure S5: Energy diagram for the ozone ($O_3$) addition to carbon-carbon bond in mmen-$Mg_2$(dobpdc). The pre-reaction complex (Image 0) and the intermediate product (Image 6) are connected through the transition state, Image 4. Relative energy values (kcal/mol) were determined using DFT calculations. $\Delta E$ is the DFT electronic energy difference between transition state and pre-reaction complex.

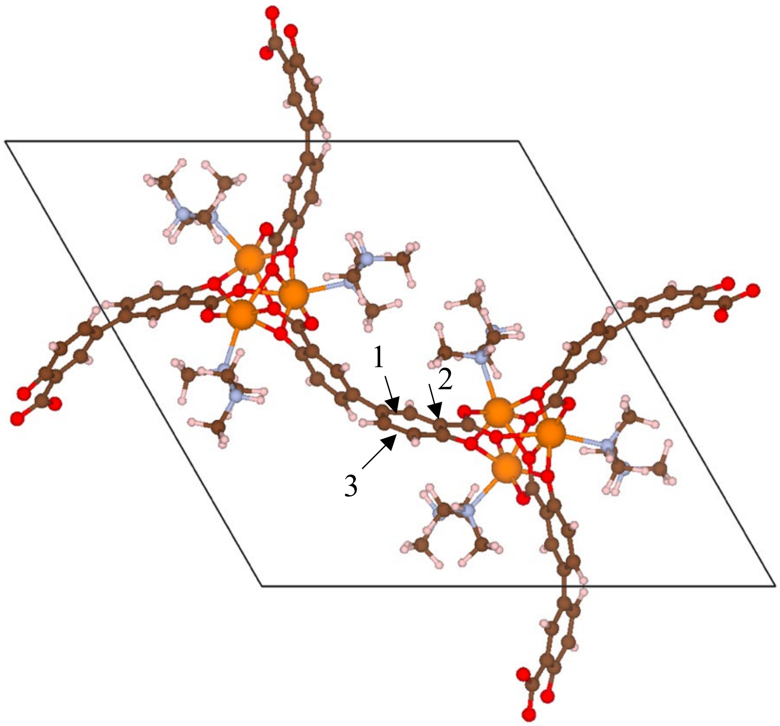


Figure S6: Unit cell of mmen-$Mg_2$(dobpdc) depicting three types of carbon-carbon double bonds in linker for possible ozone addition
